# Improved Thermophysical and Mechanical Properties in LiNaSO_4_ Composites for Thermal Energy Storage

**DOI:** 10.3390/nano14010078

**Published:** 2023-12-27

**Authors:** Maria Taeño, Ariba Adnan, Cristina Luengo, Ángel Serrano, Jean-Luc Dauvergne, Paola Crocomo, Ali Huerta, Stefania Doppiu, Elena Palomo del Barrio

**Affiliations:** 1 Center for Cooperative Research on Alternative Energies (CIC Energigune), Basque Research and Technology Alliance (BRTA), Alava Technology Park, Albert Einstein 48, 01510 Vitoria-Gasteiz, Spain; aribaadnan50@gmail.com (A.A.); pcrocomo@cicenergigune.com (P.C.); epalomo@cicenergigune.com (E.P.d.B.); 2 Ikerbasque, Basque Foundation for Science, 48013 Bilbao, Spain

**Keywords:** solid-solid PCMs, mechanical properties, thermal energy storage, MgO, EG600, thermal conductivity

## Abstract

Solid-solid phase-change materials have great potential for developing compact and low-cost thermal storage systems. The solid-state nature of these materials enables the design of systems analogous to those based on natural rocks but with an extraordinarily higher energy density. In this scenario, the evaluation and improvement of the mechanical and thermophysical properties of these solid-solid PCMs are key to exploiting their full potential. In this study, LiNaSO_4_-based composites, comprising porous MgO and expanded graphite (EG) as the dispersed phases and LiNaSO_4_ as the matrix, have been prepared with the aim of enhancing the thermophysical and mechanical properties of LiNaSO_4_. The characteristic structure of MgO and the high degree of crystallinity of the EG600 confer on the LiNaSO_4_ sample mechanical stability, which leads to an increase in the Young’s modulus (almost three times higher) compared to the pure LiNaSO_4_ sample. These materials are proposed as a suitable candidate for thermal energy storage applications at high temperatures (400–550 °C). The addition of 5 wt.% of MgO or 5% of EG had a minor influence on the solid-solid phase change temperature and enthalpy; however, other thermal properties such as thermal conductivity or specific heat capacity were increased, extending the scope of PCMs use.

## 1. Introduction

The use of phase change materials (PCMs) as thermal energy stockpiling storage media has been a subject of intense research in the last decades [1,2,3]. This research has been focused on PCMs [4,5] with melting points at low/medium temperatures, although anhydrous salts and metal alloys have also been studied for storage applications at high temperatures. Compared to sensible heat storage, PCMs bring several advantages, such as higher energy density and energy storage at almost constant temperature, whereas they lead to less expensive storage solutions when compared to thermochemical storage [6,7,8]. Solid-liquid PCMs undergoing melting/solidification processes are the most used PCMs so far because they usually have a much higher enthalpy of phase change than PCMs undergoing solid-state phase transitions (solid-solid PCMs). However, they suffer from leakage and usually have low thermal conductivity, thus requiring expensive heat exchangers or macro-encapsulation for their implementation into the storage system. In this sense, the use of solid-solid PCMs would have the advantage of not requiring either macro-encapsulation or heat exchangers, thus reducing considerably the cost of the thermal storage system. A comprehensive overview of solid-solid PCMs can be found in recent reviews by Fallahi et al. [9] and Usman et al. [10]. 

A suitable solid-solid PCM must not only have a sufficiently high phase transition enthalpy and high thermal conductivity, among others, but it also needs to be stable upon cycling (heating/cooling cycles), preferably compatible with air, and mechanically stable. As previously reported, the use of solid-solid transitions based on LiNaSO_4_ has a great potential for thermal energy storage applications [10,11,12].

Although the thermal properties of this material, such as the transition enthalpy and entropy, the specific heat capacity, the thermal conductivity [13], or the thermal expansion [14], have been reported, to the best of our knowledge, the mechanical behavior of this compound, as well as the attempts to improve the thermophysical properties, have not been previously reported and discussed. Since thermal conductivity and mechanical behavior, among other aspects, are key factors regarding the application of these PCMs as thermal energy storage (TES) materials, enlightened understanding is needed.

These inorganic materials usually have low thermal conductivity (0.5–0.8 W/mK [15,16]), which reduces their thermal energy storage performance. Therefore, an enhancement of thermal conductivity is important for the development of efficient thermal energy storage systems. Numerous researchers have dealt with the improvement of the thermal conductivity of inorganic PCMs by the addition of highly conductive nanostructures [3,17]. It was found that the improvement depends on their size, shape, or concentration, among others. Magnesium oxide (MgO), aluminum oxide (Al_2_O_3_), copper (Cu), copper oxide (CuO), gold (Au), silver (Ag), titanium oxide (TiO_2_), zinc oxide (ZnO), or expanded graphite (EG) are the most commonly used nanostructures employed for thermal energy storage applications [18,19,20,21,22]. From a mechanical point of view, the use of porous ceramics such as MgO or Al_2_O_3_, together with an increase in thermal conductivity, also enhances the mechanical strength of the pure PCMs [23,24]. For example, C. Li et al. [24] studied the influence of the addition of MgO with different particle sizes to an inorganic PCM, concluding that smaller MgO particles yielded smaller internal pores and a more rigid structure. Taking this into account, the addition of binders at macro/microscales can provide many benefits since thermal conductivity and mechanical strength can be enhanced simultaneously, extending the scope of PCM use. 

Since the implementation of this PCM in thermal energy systems usually requires an improvement of the thermophysical and mechanical properties of the pure material, in this work the influence of the addition of different binders (carbon-based and ceramic-based) was investigated. A special focus is given to the study of the mechanical and thermophysical properties of the pure LiNaSO_4_ stoichiometric compound and the influence of the addition of binders such as the synthesized nanoporous MgO and the Expanded Graphite (EG600). 

## 2. Materials and Methods

### 2.1. Theoretical Aspects

**Figure 1:**
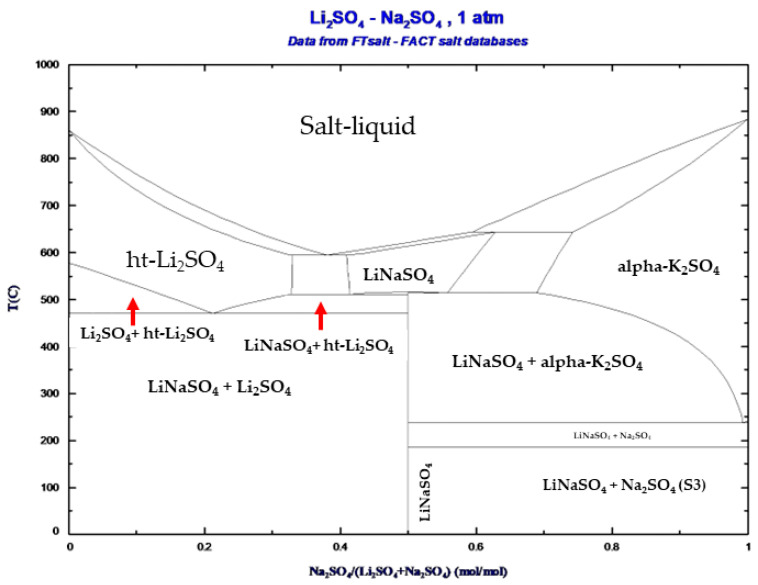
The theoretical phase diagram was obtained using the FactSage software.

The theoretical phase diagram of Li_2_SO_4_-Na_2_SO_4_ obtained using the FactSage 8.2 software [25] is shown in Figure 1. The CALPHAD method predicts the solid-solid phase transition at the composition Li_2_SO_4_-Na_2_SO_4_ 50/50 (molar ratio). 

### 2.2. LiNaSO_4_ Stoichiometric Mixture Preparation 

Li_2_SO_4_ and Na_2_SO_4_ anhydrous powders were supplied by Alfa Aesar with purities of 99.7% and 99%, respectively. In order to avoid the hydration of Li_2_SO_4_, the pure materials were placed in an Argon glove box (MBRAUN) with levels of oxygen and humidity lower than 0.1 ppm. The pure materials (Li_2_SO_4_ and Na_2_SO_4_) were mixed in the proper stoichiometric molar ratio (50/50) and subjected to a mechanical treatment (ball milling). For this purpose, a Spex mixer mill (875 RPM, SpexSamplePrep, Metuchen, NJ, USA) with stainless steel vials and balls was used. The mechanical treatment was performed for 15 min using seven balls of one gram (ball-to-powder mass ratio 1.4) to obtain a homogeneous mixture with a good intermixing degree. The resulting powder mixture was thus subjected to a thermal treatment in an oven (Nabertherm) up to 550 °C for 15 min with a heating and cooling rate of 10 °C/min to obtain the stoichiometric compound (hereafter referred to as LNS_50:50).

### 2.3. Synthesis of Binders and LNS-Binder Composites Preparation 

Four different composites were synthesized, corresponding to the LNS_50:50 stoichiometric compound with expanded graphite (EG600), the LNS_50:50 stoichiometric compound with magnesium oxide (MgO), the LNS_50:50 stoichiometric compound with aluminum oxide (Al_2_O_3_), and the LNS_50:50 stoichiometric compound with zirconium oxide (ZrO_2_). In the case of composites containing EG600, Al_2_O_3_, and ZrO_2_ as binders, commercial powders were employed. Expanded graphite (EG600) was purchased from SIGRATHERM, Madrid, Spain (Batch 347/11/16), Al_2_O_3_ was purchased from Sigma-Aldrich, St.Louis, MA, USA (≥99%), and ZrO_2_ was purchased from Sigma-Aldrich (99%). In the case of the composite containing MgO as a binder, the MgO was previously synthesized to obtain a material with a controlled morphology. For this purpose, 1.28 g of Mg(NO_3_)_2_·6H_2_O (Sigma Aldrich, ≥99%) was dissolved in 4 mL of deionized water. After the addition of 4 mL of ethylene glycol, the solution was stirred continuously for 30 min. The final product was then annealed at 600 °C for 2 h.

The composites were synthesized as follows: 5 wt.% of EG600, MgO, Al_2_O_3_, or ZrO_2_, respectively, were added to the LNS_50:50 stoichiometric compound previously synthesized and subsequently ball milled for 15 min using seven balls of one gram (ball to powder mass ratio 1.4) to obtain a homogeneous mixture. The powders were then pressed for 15 min at 3 tons in the form of pellets (1 g) using a pellet die of 13 mm and subjected to a thermal treatment of 550 °C for 10 h.

### 2.4. Selection of Binders

The selection of the most promising binders was performed based on the enthalpy values. Table 1 shows the values of the enthalpies of the solid-solid phase transition for all the composites. The values for the stoichiometric compound LNS_50:50 have been added as references.

In view of the results, the samples containing Al_2_O_3_ and ZrO_2_ showed a clear decrease in the enthalpy values. Moreover, the enthalpy values corresponding to the composites containing MgO and EG600 as binders agree with those observed for the pure LNS_50:50, as can be observed in Table 1. Taking all these results into account, in this work, two different binders, EG600 and MgO, were selected in order to improve the mechanical stability and thermal conductivity of the pure material. 

### 2.5. Structural, Morphological, Thermophysical and Mechanical Characterization

The structural characterization of the materials was performed by X-ray diffraction analysis using a Bruker D8 Discover equipped with a LYNXEYE XE detector with monochromatic Cu Kα1 radiation of λ = 1.54056 Å. XRD patterns were recorded in a 2θ angular range of 10–80° with a step size of 0.02° and a step time of 0.96 s. The measurements were performed at room temperature. A profile-fitting procedure of the diffraction patterns based on the Rietveld method (FullProf software [26]) was used to identify the presence of different phases. The morphology of the materials was studied by scanning electron microscopy (SEM) using a QUANTA 200 FEG (FEI Company, Hillsboro, OR, USA) microscope operated in high vacuum mode at 5 kV and with a backscattered electron detector (BSED). 

The reactivity of the materials was studied by differential scanning calorimetry (DSC) using a Discovery DSC Q2500 model (TAinstruments, New Castle, PA, USA) with an accuracy of ±0.04%. This technique allows for the determination of the enthalpies of the solid-solid phase transition, the transition temperatures, and the specific heat capacities. Before recording the DSC signals, all the samples were subjected to a compatibility test in an oven (inside hermetically closed DSC aluminum crucibles) in order to detect any unwanted side reactions or evaporation of the material. Three DSC heating/cooling cycles were performed for each sample in the temperature range of 25 °C to 550 °C using a heating and cooling rate of 5 °C/min. The specific heat capacity measurements were performed on the same equipment using a modulated heating ramp dynamic method [27]. For these measurements, the instrument was previously calibrated using sapphire as the standard material. 

The transient Hot Disk method was used to determine the thermal conductivity of each sample as described in [28] and following Hot Disk ISO 22007-2:2022 [29]. For these tests, two 13 mm-diameter cylindrical pellets were used for each tested sample. For each sample, a series of 6 experiments of 20 s were performed at 30 °C, 50 °C, and 100 °C with a TPS 2500 instrument (Hot Disk, Ghotenburg, Sweden) equipped with a Kapton insulated sensor of 2 mm diameter (sensor type 7577F1). 

The textural properties of MgO were characterized using an automated gas adsorption analyzer (Micrometrics ASAP 2460, Norcross, GA, USA). The nitrogen sorption curve of the material was measured under isothermal conditions after outgassing at 200 °C in a vacuum for 5 h. The multipoint surface area was evaluated with the Brunauer–Emmet–Teller (BET) method, and the pore size distribution was obtained using the Barret–Joyner–Halenda (BJH) model applied to the desorption isotherm branch. The bulk density of the samples was calculated considering the dimensions and the mass of the pellets, while the real density was measured using a Helium pycnometer (AccuPyc II 1340, Micromeritics, Norcross, GA, USA). 

Finally, compression tests were performed in order to determine the mechanical behavior of the samples. For this purpose, an Instron 5697 device (Instron, Norwood, MA, USA), provided by a load cell of 3000 N in compression mode, was employed. The experiments were performed using a crosshead speed of 1 mm/min. All the specimens (five pellets for each analyzed material with dimensions of 10 mm × 10 mm) were subjected to a pressure of 3 tons for 15 min at room temperature.

## 3. Results and Discussion

**Figure 2:**
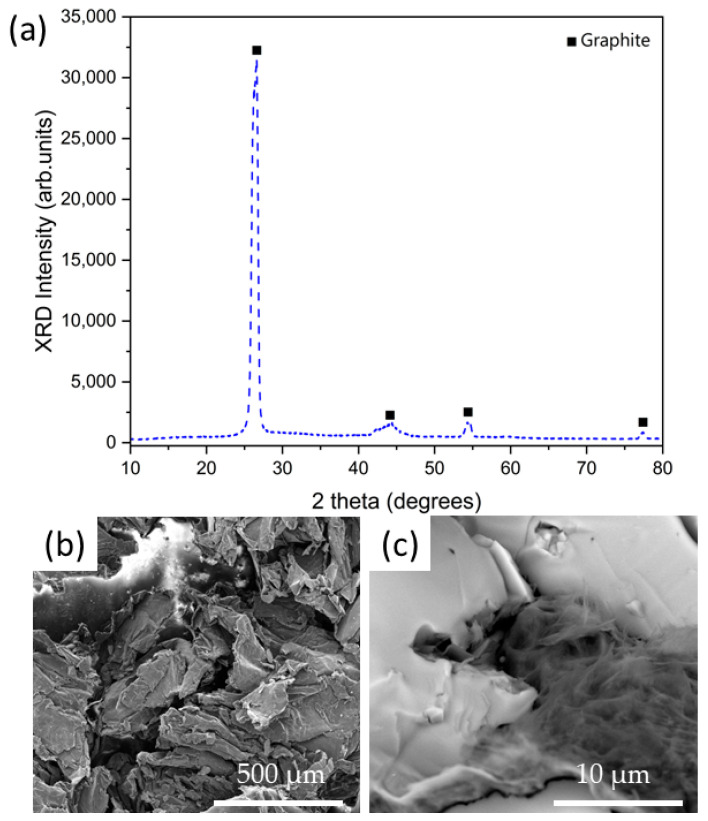
(**a**) (XRD) pattern for EG600 commercial powder. (**b**,**c**) SEM images acquired at low and high magnifications for EG600 commercial powders.

The materials employed as binders (EG600 and MgO) were structurally and morphologically characterized at first. Figure 2a shows the XRD pattern for commercial EG600 powder, while Figure 2b,c show the corresponding SEM images acquired at low and high magnifications. Figure 2a shows four main peaks centered at 26.5°, 44.2°, 54.4°, and 77.3° corresponding to the graphite structure (ICSD 76767), confirming the crystalline nature of the EG600 commercial powders. Figure 2b,c show the SEM images acquired for the pure EG600 sample. In the case of the image acquired at low magnifications (Figure 2b), a porous surface with small voids and cracks can be observed, while the image acquired at high magnifications (Figure 2c) shows the characteristic morphology of the expanded graphite, composed of sheets of graphite.

**Figure 3:**
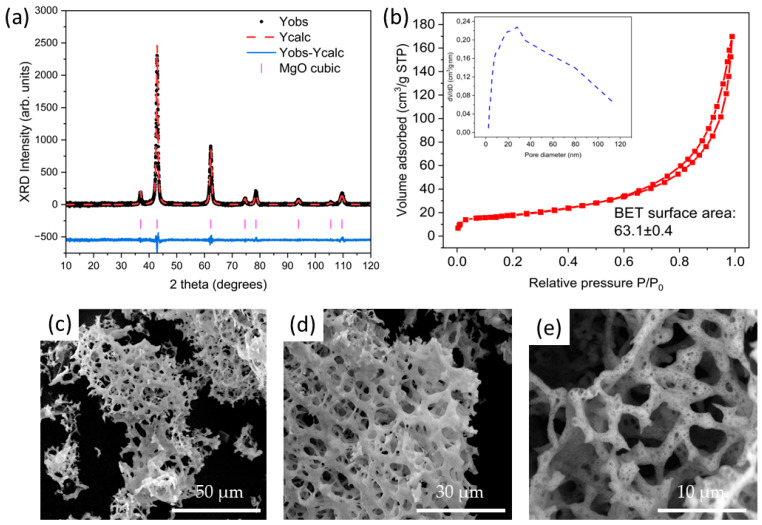
(**a**) XRD results and refinement for the MgO sample. (**b**) Nitrogen adsorption-desorption curves and pore size distribution for the MgO sample; (**c**–**e**) SEM images acquired at different magnifications for the MgO sample.

The most relevant results for MgO are shown in Figure 3. Figure 3a shows the XRD pattern and the corresponding refinement for the MgO sample, where the diffraction peaks corresponding to the cubic structure can be indexed (ICSD 9863). Crystal sizes of 21.1 nm were obtained from the Rietveld refinement. The textural properties of this material were analyzed by gas adsorption curves, obtaining a BET surface area of 63.1 m^2^/g, as can be observed in Figure 3b. Moreover, the pore size distribution is shown in the inset of Figure 3b, where the typical size of the pores is 30 nm. The surface area obtained in this work is similar to other works in literature. It is well known that this parameter is highly dependent on the synthesis method, precursors, and annealing conditions. For example, S. Alai et al. [30] reported BET surface areas ranging from 69.8 m^2^/g to 97.8 m^2^/g as a function of the ratio of the precursors, while A. L. Sadgar et al. [31] reported values from 35.09 m^2^/g to 116.7 m^2^/g as a function of the annealing conditions. The morphology of the synthesized MgO was studied by means of SEM. Figure 3c–e, show the SEM images acquired at different magnifications where pure MgO with a high degree of porosity can be observed. In this case, three-dimensional networks formed by MgO can be observed. In addition to the three-dimensional networks observed in the high-magnification images, nanometric pores can be seen in Figure 3e, confirming the pore size obtained from the analysis of the desorption curves. 

**Figure 4:**
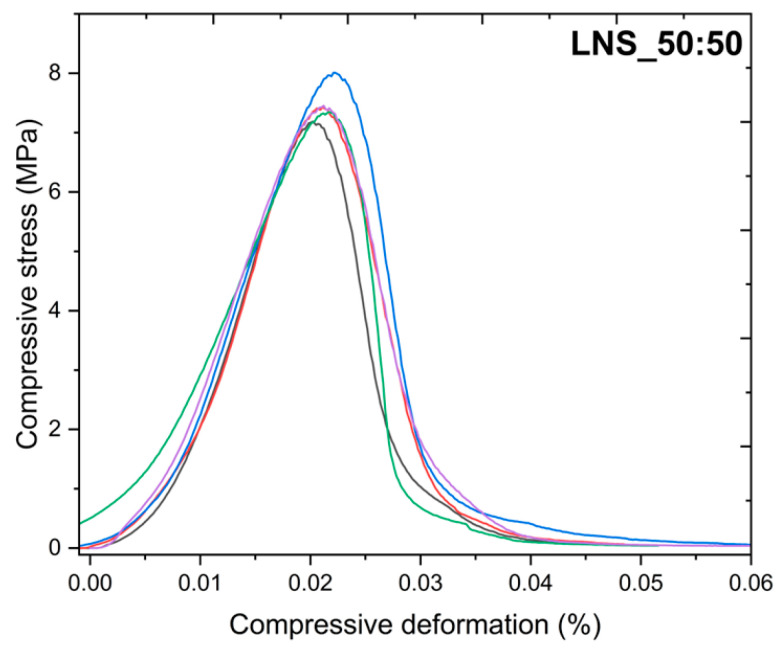
Compressive deformation vs. compressive stress curves for the LNS_50:50 sample.

As already mentioned in the introduction, the mechanical properties of the LiNaSO_4_ stoichiometric compound have not been previously reported. Therefore, one of the aims of this work is to study the behavior of this material under compression tests. For this purpose, as described in the experimental section, five specimens of each sample with dimensions of 10 mm × 10 mm were prepared. Figure 4 shows the compressive deformation vs. compressive stress curves for the pure LNS_50:50 stoichiometric compound.

As can be observed, the curves for the five specimens are similar and show good reproducibility. By analyzing the slope of each of the deformation-stress curves, the Young’s modulus can be easily calculated. The corresponding average values for the Young’s modulus and the yield point are 494 MPa and 7.5 MPa, respectively. Since this is the first time that the mechanical properties of this material have been reported, there is no possible comparison with the data in the literature. However, some works regarding the mechanical properties of nitrate thermal storage salts in the solid phase can be found in the literature. B. D. Iverson et al. reported Young’s modulus values of three different salts (solar salt, HITEC salt, and Na-Ca-Li-K nitrate salt) ranging from 3 GPa to 20 GPa. It should be noted that the mechanical properties reported by B. D. Iverson were performed as a function of temperature, while the experiments carried out in this work were performed at room temperature; therefore, a direct comparison cannot be made. Although the Young’s modulus values reported here are lower, a clear enhancement of the mechanical properties has been demonstrated with the addition of binders, which confirms that there is still room for improvement.

Taking these results into account, the implementation of this material as a phase-change material for thermal energy storage applications usually requires an enhancement in its mechanical properties. For this reason, the influence of the addition of the binders selected on the structural, thermophysical, and mechanical properties of the LNS_50:50 stoichiometric compound was studied.

**Figure 5:**
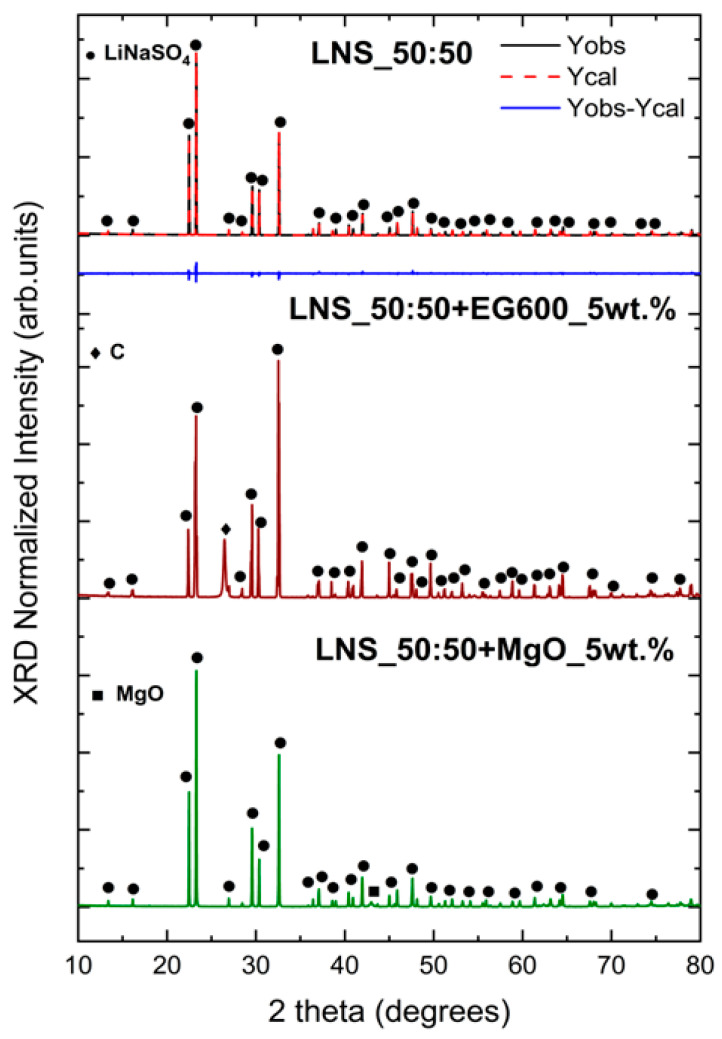
XRD patterns for LNS_50:50 pure sample, LNS_50:50+EG600_5wt.%. and LNS_50:50+MgO_5wt.%.

As a first step, in order to confirm that the addition of EG600 and MgO does not alter the structural properties of the pure LNS_50:50 stoichiometric compound, X-ray diffraction analysis was carried out after the synthesis of the composites (see Figure 5). The XRD of the pure LNS_50:50 and the corresponding Rietveld refinement are also reported in Figure 5 for comparison.

The XRD analysis confirms the presence of the LiNaSO_4_ stoichiometric compound (ICSD 14364) [11,32] in the three samples. Moreover, the XRD pattern of the LNS_50:50+EG600_5wt.% sample shows the most intense peak of the graphite phase (ICSD 76767) at 26.4°, while in the XRD pattern of the LNS_50:50+MgO_5wt.% sample, the most intense peak of MgO (ICSD 9863) at around 43° is evident. The relative intensity of these peaks is, as expected, due to the low amount of binder added, considerably lower than in the LiNaSO_4_ peaks. In view of these results, it can be confirmed that the incorporation of both binders does not cause the formation of unwanted secondary phases, suggesting the chemical compatibility of the materials.

**Figure 6:**
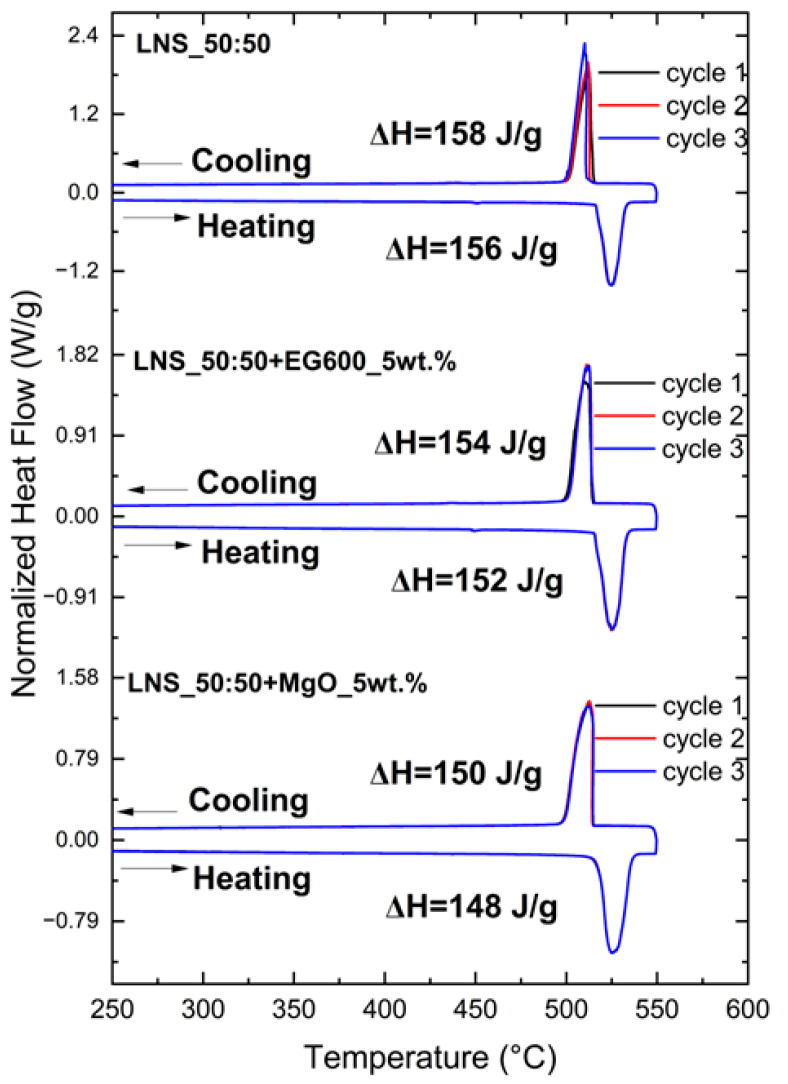
DSC curves for LNS_50:50, LNS_50:50+EG600_5wt.%, and LNS_50:50+MgO_5wt.%, including the enthalpy values.

This is confirmed by the DSC results shown in Figure 6. As described in the experimental section, the samples containing the binders were already subjected to a thermal treatment at 550 °C for 30 min before the DSC measurements.

The results show that the addition of binders does not affect the stoichiometry of the pure LNS_50:50. Moreover, all the samples are thermally stable since there are no significant variations among the three cycles. The energy corresponding to the solid-solid transition for the pure LNS_50:50 is 156 J/g, in agreement with the experimental values previously reported [11,33,34]. The enthalpy values for the samples containing the binders are, as expected, slightly lower (152 J/g for LNS_50:50+EG600 and 148 J/g for LNS_50:50+MgO) than for the pure LNS_50:50 due to the inert additives (5 wt.%). Furthermore, no significant differences in the transition temperature (around 518 °C for all the samples) and no apparent subcooling were observed. Table 2 shows the average of the enthalpy values (considering the three cycles) with a standard deviation lower than 2% for all the measurements.

In addition, the specific heat capacities for all the samples were measured in the DSC apparatus (see experimental section), and the corresponding values are shown in Table 2. Three repetitions were carried out for each of the samples, and the values were generally within ±5% error, being the results within the measurement accuracy of the DSC. Table 2 shows the specific heat capacity for all three samples at 540 °C, where the α-LiNaSO_4_ cubic phase is formed. These values are lower than those measured at 500 °C (before the solid-solid transition where the trigonal phase is formed), being 1.81, 1.76, and 1.68 J/g·K for pure LNS_50:50, LNS_50:50+EG600_5wt.%, and LNS_50:50+MgO_5wt.%, respectively. The theoretical value for the pure LiNaSO_4_ cubic phase is 1.50 J/g·K at 500 °C, according to data from FactSage 8.2, which agrees with the experimental value measured in this work and the experimental value reported by A. Bayon et al. [33]. However, the addition of binders leads to an increase in the specific heat capacities, as can be observed in Table 2. The increased values might be attributed to the large specific heat capacities of the EG600 and MgO powders. The increase of this parameter with the addition of MgO has been previously reported [35,36]. For example, Y. Huang et al. [36] calculated that the increase in specific heat capacity of the modified salt is not only related to the properties of nano-MgO but also to the adsorption of nanoparticles on the salt where the solid layer is formed. 

It is well known that in order to consider a PCM as a good candidate for thermal energy storage applications, some requirements need to be covered. Apart from a high specific heat capacity or high enthalpy of transition, as already demonstrated in this work, a high thermal conductivity is necessary to improve heat transfer. For this reason, thermal conductivity measurements have been performed at 100 °C, where the LiNaSO_4_ trigonal structure is stable, and are shown in Table 2. Pure LNS_50:50 compound shows a thermal conductivity of 0.47 W/m·K, in agreement with those reported by B. M. Suleiman et al. [13] at this temperature. A clear increase in the thermal conductivity values with the addition of binders can be observed, more evident in the case of the LNS_50:50+EG600_5wt.% sample. It is well known that EG exhibits a high thermal conductivity [37,38,39], which is one of the main reasons for its use as an additive in thermal energy storage applications. Y. Zhang et al. [38] studied the influence of the different additives on the thermal conductivity of shape-stabilized phase change materials, confirming that among all the analyzed samples, the one with EG has the largest thermal conductivity. Similar results were reported by A. Mills et al. [39], where the addition of EG leads to thermal conductivity values 20–130 times higher than the thermal conductivity of pure PCM. In this work, the addition of EG600 as binder leads to a thermal conductivity around three times higher than the pure LNS_50:50, while the thermal conductivity value for the sample containing MgO as binder is only slightly higher than the pure LNS_50:50. Few works in the literature report the addition of MgO to improve the thermal conductivity of PCMs. For example, M. K. Saranprabhu et al. [35] reported that the solid-phase thermal conductivity of MgO-solar salt was increased by 17.5% with a dispersion of 0.25 wt.% MgO nanoparticles. In addition, the authors studied the influence of the nanoparticle’s concentration on thermal conductivity, showing that increasing the additive concentration does not always lead to a thermal conductivity enhancement. 

It is well known that some parameters, such as the incorporation of binders [40], porosity [41], grain size [42,43], or density [44], strongly affect the mechanical behavior of the final materials. For this reason, before the study of the mechanical properties of the composites, the bulk and real density, together with the total porosity, were measured for all the samples (shown in Table 3), as well as the morphology of the composites by means of SEM. For the total porosity calculation, the theoretical value of the density of pure stoichiometric LiNaSO_4_ (FactSage 8.2 software) has been taken as a reference (2.458 g/cm^3^). 

The bulk density values of the three analyzed samples show significant differences, as can be observed in Table 3. In particular, the sample containing MgO shows the highest values and the lowest porosity value, as expected. Porosity has been known to have a strong effect on the mechanical properties of materials, often leading to their weakening. Highly porous materials, such as MgO and EG600, sometimes prove troublesome for supporting load-based structures due to the presence of voids in the microstructure of the material. 

**Figure 7:**
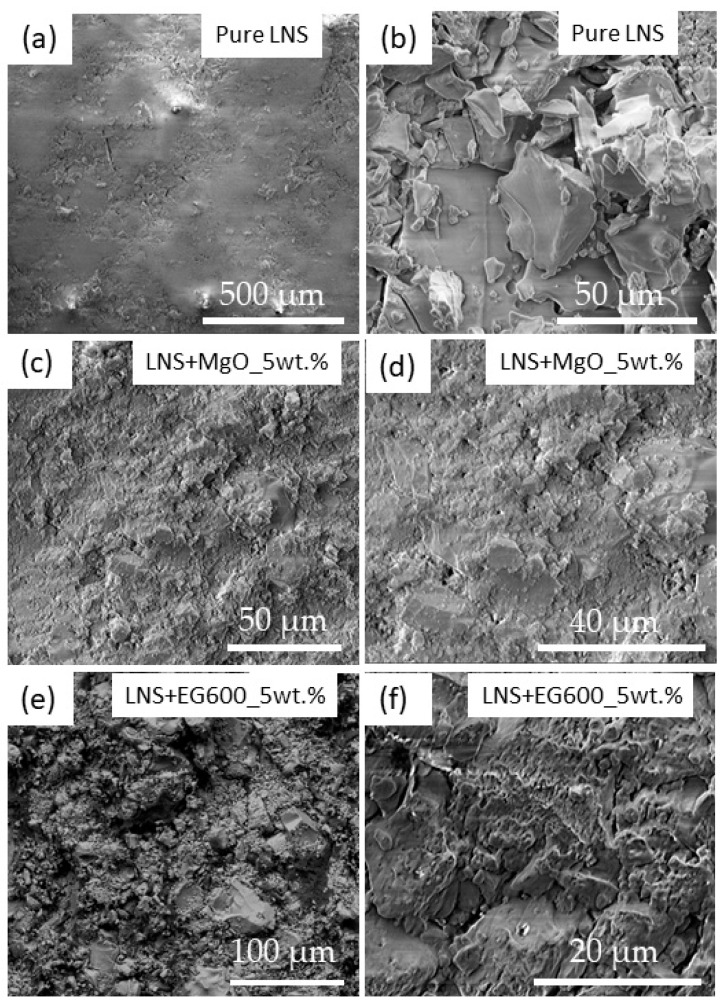
SEM images acquired for: (**a**,**b**) pure LNS sample; (**c**,**d**) LNS+MgO_5wt.% sample; (**e**,**f**) LNS+EG600_5wt.% sample.

In order to gain a deeper understanding of the values of the porosity and density shown in Table 3 and correlate them later with the mechanical properties, the microstructure of the three samples has been studied by means of SEM. The SEM images of the pure LNS_50:50 have been added as references (see Figure 7). A smooth and homogeneous surface corresponding to the presence of the LiNaSO_4_ stoichiometric compound can be observed in Figure 7a. Small regions of the surface exhibit agglomerated grains of tens of microns, as shown in Figure 7b. As expected, the pure LNS_50:50 does not show a high porosity (which confirms the values reported in Table 3), although small voids and cracks can be observed in specific regions through the surface. Taking all this information into account, the SEM images for the composites containing MgO and EG600 have been acquired. Figure 7d shows the SEM image of the LNS_50:50+MgO_5wt.% sample, where small and agglomerated particles covering the analyzed surface can be observed. The characteristic three-dimensional networks previously observed (Figure 3) for pure MgO have disappeared, probably due to the ball milling process employed for the preparation of the LNS_50:50+MgO_5wt.% sample. The presence of these small particles over the surface confers on the sample a homogeneous morphology where the presence of pores, voids, or cracks is not detected. SEM images for the LNS_50:50+EG600_5wt.% sample are shown in Figure 7e,f. In this case, a more porous structure has been obtained, as shown in Figure 7f, where the expanded graphite is mainly covering the grains of LNS_50:50, similar to those observed in Figure 7b. These results agree with the porosity values shown in Table 3, where the LNS_50:50+EG600_5wt.% sample is the one with the highest porosity.

**Figure 8:**
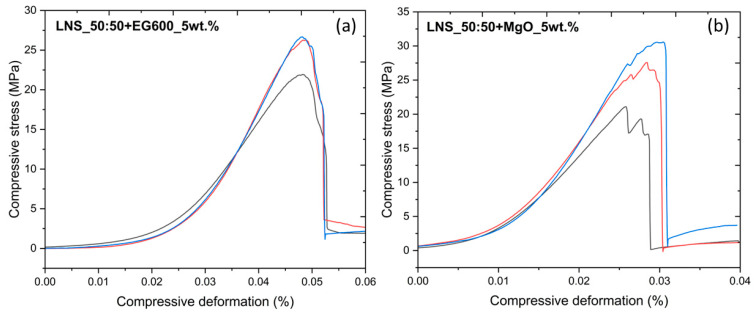
Compressive deformation vs. compressive stress curves for (**a**) LNS_50:50+EG600_5wt.% and (**b**) LNS_50:50+MgO_5wt.%.

As previously mentioned, one of the objectives of this work is to enhance the mechanical properties of the pure LNS_50:50. For this purpose, similar compression tests, as previously described, were carried out on the samples containing EG600 and MgO. Figure 8a shows the compressive deformation vs. compressive stress curves for the LNS_50:50_EG600_5wt.%, while Figure 8b shows the compressive deformation vs. compressive stress curves for the LNS_50:50_MgO_5wt.%. All the measurements with an error higher than 10% were discarded. To this end, Figure 8a,b only shows the compressive deformation vs. compressive stress curves for the experiments with the minimum error.

Following the procedure previously described, the corresponding average values for the Young’s modulus and the yield point are shown in Table 4 (values for LNS_50:50 have been added as references). It should be noted that the average values shown in Table 4 for the Young’s modulus of the different samples confirm the good reproducibility of the measurements. However, a clear dispersion in the values of the yield point can be observed; therefore, the average values are considered only for comparison with the pure PCM. The results show a drastic increase in both Young’s modulus and yield points for the two composites. A higher Young’s modulus value implies that the material is stiffer, and therefore the deformations will be smaller. The enhancement of the mechanical properties of the inorganic PCMs with the addition of EG or MgO has been previously reported by several authors [45,46,47]. For example, M. Li et al. [48] reported that carbon-based materials have a spatial network structure, which causes an increase in the contact areas with the PCM, which is beneficial to enhancing the mechanical properties of PCMs. On the other hand, Q. Li et al. [47] demonstrated that the higher surface energy of nano- and porous-MgO induced high particle rearrangement, coarsening, and composite densification, which contributed to the improvement of the mechanical properties of PCMs. 

It should be noted that LNS_50:50+MgO_5wt.% is the sample with the highest Young’s modulus and yield point and with the lowest total porosity (see Table 3). This behavior is the expected one, considering the relationship between porosity and Young’s modulus [49]. Nevertheless, this relationship cannot be considered for the LNS_50:50+EG600_5wt.% sample since it exhibits a higher Young’s modulus than the pure LNS_50:50 and the highest total porosity value. Despite the high porosity of the EG [50,51], it is well known that this material exhibits a high degree of crystallinity, as observed by XRD, which confers on the final material greater structural stability, which could be responsible for the high Young’s modulus observed in comparison with the pure LNS_50:50. 

## 4. Conclusions

Summarizing different aspects should be taken into account:

- A LiNaSO_4_ stoichiometric compound containing MgO and EG600 as binders was successfully synthesized following a simple and rapid ball milling process.
- The pure stoichiometric compound as well as the composites containing porous MgO and EG600 were deeply characterized by structural, thermal, and mechanical techniques. A clear enhancement in the thermophysical and mechanical properties of LiNaSO_4_ was demonstrated by adding 5 wt.% of MgO and EG600, respectively.
- The studied composites exhibited high thermal and chemical stability since no side reactions or decrease in storage density were observed.
- The thermal conductivity of the stoichiometric LiNaSO_4_ was enhanced almost three times with the addition of EG600. This enhancement of the thermophysical properties of the samples containing the binders was accompanied by an enhancement of the mechanical properties.
- The Young’s modulus increased three times with the addition of MgO porous structure in comparison to the pure stoichiometric compound, which could potentially open the field of application of this material.

These results confirm that these composites can be considered promising candidates for thermal energy storage applications; however, further steps need to be taken, such as the study of their long-term stability. To this end, the cyclability of these materials will be studied in depth in future work, investigating the thermophysical and mechanical properties of the composites after 100 cycles.

## Figures and Tables

**Table 1:** Enthalpy values and temperatures for the pure LNS_50:50 and the different binders.

Sample	${∆H}_{heating}$ (J/g)	${∆H}_{cooling}$ (J/g)
LNS_50:50	156	158
LNS_50:50+EG600_5wt.%	152	154
LNS_50:50+MgO_5wt.%	148	150
LNS_50:50+Al_2_O_3__5wt.%	129	140
LNS_50:50+ZrO_2__5wt.%	110	138

**Table 2:** Enthalpy values from DSC heating and cooling cycles, specific heat capacities, and thermal conductivity at 100 °C for all the analyzed samples.

Sample	${∆H}_{heating}\;(J/g)$			${∆H}_{cooling}\;(J/g)$			$c_{p}\;(J/g\cdot K)$	k (W/m·K)
	Ons Temp. (°C)	Off Temp. (°C)	Peak Temp. (°C)	Ons Temp. (°C)	Off Temp. (°C)	Peak Temp. (°C)		
LNS_50:50	156			158			1.60	0.47 ± 0.01
	517	535	524	514	502	513		
LNS_50:50+EG600_5wt.%	152			154			1.62	1.25 ± 0.04
	517	536	525	514	499	511		
LNS_50:50+MgO_5wt.%	148			150			1.67	0.49 ± 0.01
	518	539	525	514	496	513		

**Table 3:** Bulk density, real density, and porosity measurements for LNS samples.

Sample	$\rho_{bulk}$ (g/cm^3^)	$\rho_{real}$ (g/cm^3^)	Total Porosity (%)
LNS_50:50	2.003 ± 0.004	2.549 ± 0.005	18.5
LNS_50:50+EG600_5wt.%	1.906 ± 0.004	2.581 ± 0.013	22.4
LNS_50:50+MgO_5wt.%	2.019 ± 0.004	2.602 ± 0.005	17.8

**Table 4:** Average values of Young’s modulus and yield point for pure LNS_50:50, LNS+EG600_5wt.%, and LNS+MgO_5wt.%.

Sample	Young’s Modulus (MPa)	Yield Point (MPa)
LNS_50:50	494	7.5
LNS_50:50+EG600_5wt.%	1177	24.9
LNS_50:50+MgO_5wt.%	1508	24.7

## Data Availability

Data are contained within this article.
